# In Silico Identification of Antimicrobial Peptides in the Proteomes of Goat and Sheep Milk and Feta Cheese

**DOI:** 10.3390/proteomes7040032

**Published:** 2019-09-21

**Authors:** Marios Tomazou, Anastasis Oulas, Athanasios K. Anagnostopoulos, George Th. Tsangaris, George M. Spyrou

**Affiliations:** 1 The Cyprus Institute of Neurology & Genetics, Bioinformatics Group, 6 International Airport Avenue, 2370 Nicosia, Cyprus, P.O. Box 23462, 1683 Nicosia, Cyprus; mariost@cing.ac.cy (M.T.); anastasioso@cing.ac.cy (A.O.); 2 The Cyprus School of Molecular Medicine, 6 International Airport Avenue, 2370 Nicosia, Cyprus, P.O. Box 23462, 1683 Nicosia, Cyprus; 3 Proteomics Research Unit, Biomedical Research Foundation of the Academy of Athens, 115 27 Athens, Greece; atanagnost@bioacademy.gr (A.K.A.); gthtsangaris@bioacademy.gr (G.T.T.)

**Keywords:** peptidomics, proteomics, antimicrobial peptides, milk whey, proteolysis, proteases, in silico digestion, functional foods, gastrointestinal tract

## Abstract

Milk and dairy products are a major functional food group of growing scientific and commercial interest due to their nutritional value and bioactive “load”. A major fraction of the latter is attributed to milk’s rich protein content and its biofunctional peptides that occur naturally during digestion. On the basis of the identified proteome datasets of milk whey from sheep and goat breeds in Greece and feta cheese obtained during previous work, we applied an in silico workflow to predict and characterise the antimicrobial peptide content of these proteomes. We utilised existing tools for predicting peptide sequences with antimicrobial traits complemented by in silico protein cleavage modelling to identify frequently occurring antimicrobial peptides (AMPs) in the gastrointestinal (GI) tract in humans. The peptides of interest were finally assessed for their stability with respect to their susceptibility to cleavage by endogenous proteases expressed along the intestinal part of the GI tract and ranked with respect to both their antimicrobial and stability scores.

## 1. Introduction

A growing body of evidence suggests that milk and dairy products have unique metabolic, signalling and antimicrobial effects, beside their high nutritional content. This bioactivity is mainly mediated by peptides naturally occurring during their digestion by proteases along the gastrointestinal GI tract [1,2,3]. As shown over the last decade, such peptides mediate a broad spectrum of activities including modulation of inflammatory and immune response, signalling and metabolic processes, antihypertensive and antioxidative effects, besides acting as antimicrobial agents [3].

However, while evidence supporting the bioactive potential of milk and other food-derived peptides is accumulating, it remains unclear if the peptides of interest (a) can withstand the high proteolytic activity in the gastrointestinal tract for long enough to exert an effect before being fully degraded and (b) their permeability through the intestinal epithelium is such that they can reach the target tissue or organ at physiologically relevant concentrations. In fact, it has been suggested in a critical evaluation that di- and tripeptides can permeate the intestinal epithelium and exert a biological function; however, there is not yet convincing evidence supporting the same for longer oligopeptides [4]. On the other hand, antimicrobial peptides (AMPs) with sufficient stability with respect to proteolysis are not subject to epithelial absorption and can likely have an immediate effect on the gut microbiome. The latter could be considered as an important aspect in maintaining a healthy GI tract and controlling dysbiosis, as it was recently shown that AMPs are able to suppress the growth of opportunistic pathogens like *Helicobacter pylori* [5], *Escherichia coli and Staphylococcus aureus* [6].

AMPs are typically positively charged 12–50-amino acid (a.a.)-long oligopeptides either forming secondary structures, which include α-helixes, β-sheets, loops, or remaining as extended oligopeptides [7]. Their mechanism of action involves direct microorganism killing after penetrating and disrupting the membrane bilayer, membrane proteins or intracellular targets [8]. More recently, it has been suggested that AMPs can exert their antimicrobial effect also via immunomodulation [9], although the exact mechanisms are not entirely clear. To identify biofunctional peptides in foods, one has to consider peptides’ individual amino acid composition, charge, solubility, length, amphiphilic features and secondary structure similarity with known characterised endogenous peptides in the organism of interest as well as with peptides produced by the gut flora [8,10,11].

**Figure 1:**
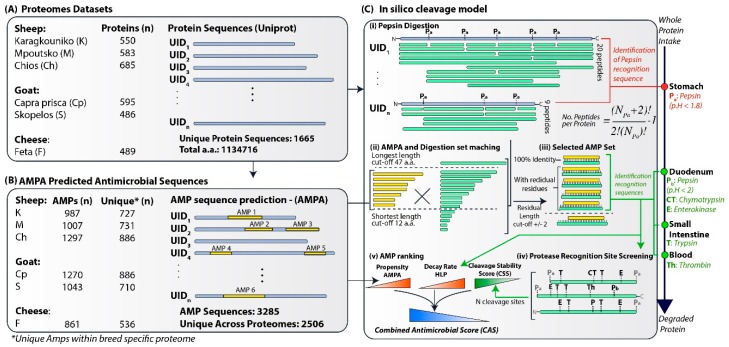
(**A**) The protein datasets analysed comprise the milk whey proteomes from three sheep and two goat breeds as well as the feta cheese proteome. (**B**) The AMPA algorithm identified the protein sequences with high antimicrobial potential with a number of antimicrobial peptides (AMPs) proportional to the initial proteome size. (**C**) The in silico cleavage analysis started by extracting all peptides occurring after pepsin digestion followed by sequence-matching with the AMPA set. The matching peptides were filtered and assessed for stability regarding their affinity to other intestinal proteases. Finally, a combined score of protease stability, half-life estimation obtained from the HLP predictor, and AMPA antimicrobial propensity was used to rank the identified peptide sequences.

Naturally, the composition of biofunctional peptides from milk and dairy products from different animal breeds is unique, offering a broad range of sequences to screen for peptides with functional traits suggesting their scientific, medical and commercial importance [7]. Milk and dairy products (e.g., yogurt) have already been characterised and identified to be effective against specific pathogens [2,12]. Sheep and goat milk was found to be rich in biofunctional peptides sourced mainly from α-, β- and k- caseins [13]. A relatively less explored proteome space to probe for AMP is represented by milk whey [14,15,16] (non-casein-rich phase) and by fermented milk products like cheese [17,18]. In this work, we focused on the potential antimicrobial properties of milk whey from two goat and three sheep pure breeds endogenous in Greece and of feta cheese, to probe for AMPs following an assessment of their stability in an intestine-like environment. Following the computational workflow shown in Figure 1, which combines existing and newly developed approaches, we characterised the antimicrobial “load” of the proteomes of interest. As shown in Figure 1, protein sequences from each breed’s milk whey and feta cheese were screened using the publicly available tool AMPA [10,11] to find sequence stretches with predicted high antimicrobial potential (i.e., low AMPA propensity). The same protein sequences were digested in silico to identify which peptides that can actually occur in the GI tract, matched the predicted AMPA stretches. The matching peptides were further assessed for their stability using as a proxy the number of cleavage sites by human endogenous proteases. The stability assessment was complemented with their respective half-life estimation by the peptide Half-Life Predictor (HLP) [19] using its Support Vector Machine (SVM) model trained on datasets obtained from crude intestinal extracts. The final ranking of the selected peptides was based on a combined antimicrobial score (CAS) calculated as a function of (a) peptide antimicrobial propensity, i.e., the potential to penetrate bacterial membranes and (b) peptide stability i.e., the peptide survival rate within an intestine-like environment necessary to have an effect. Our work resulted in a top 100 set of AMPs which are predicted to hold the highest combined stability and antimicrobial effect.

## 2. Methods

### 2.1. Protein Datasets

Milk whey proteomic datasets were obtained from previously published work. The proteomes were identified via 1-D nanoLC–MS/MS of milk whey from three sheep (Karagkouniko (K), Mpoutsko (M) and Chios (Ch)) and two goat (*Capra prisca* (CP) and Skopelos (S)) [15] breeds that are endogenous in Greece and feta cheese (F) [18]. As shown in Figure 1A, the number of proteins per proteome ranged from 489 in feta cheese up to 685 in Chios sheep.

The corresponding whole protein sequences were retrieved from the Uniprot database [20] (https://www.uniprot.org/) in fasta format for downstream analysis. A total of 1263 unique protein sequences were downloaded.

### 2.2. Prediction of Antimicrobial Peptides

Antimicrobial peptide prediction was performed using the publicly available tool AMPA [10,11] (http://tcoffee.crg.cat/apps/ampa/do). Whole-protein sequences were ran in AMPA using the default parameter values, i.e., a propensity threshold of 0.225 and a window size of 7 a.a.. The tool returned all sequence stretches of length over 12 a.a. residues that exhibited an average propensity value below the threshold [10]. Figure 1B shows a summary of all the antimicrobial sequences detected by AMPA along with their propensity (PV), probability values and all the available information of the parent protein sequences. In order to evaluate further whether the AMPA-predicted AMPs have the ability to penetrate cellular membranes, the set was screened using the publicly available CellPPD [21,22] predictor (http://crdd.osdd.net/raghava/cellppd) using the SVM classifier with a threshold of −0.1. The full set is available in Supplemental Table S2. The CellPPD results are available in Supplemental Information Figure S1.

### 2.3. Protein Cleavage Model

Cleavage site recognition was implemented in R, a programming language for statistical computing, v3.5.2 [23], by adopting the cleavage rules as regular expressions, previously introduced in the existing tools (Peptide cutter [24] and SpirPep [25]). The first phase of the script identifies cleavage sites specific for pepsin at pH < 1.8, which is typical of the acidic stomach conditions due to HCl secretion. All peptide sequences for all pairwise combinations of the identified cleavage sites including the carboxyl and amino group residues, i.e., the first and the last position of the protein sequence, were extracted. For each protein sequence *s,* the number of extracted peptides *C_s_* is given by Equation (1):

(1) $$C_{s}=\frac{\left(N_{Pa}+2\right)!}{r!\left(N_{Pa}+2-r\right)!}-1$$

where *N_Pa_* is the number of pepsin (pH < 1.8) specific cleavage sites, and *r* = 2 for pairwise combinations.

The set of resultant peptides was filtered for sequence lengths in order to keep peptides longer than the minimum peptide length in the AMPA set (>12 a.a. residues) but shorter than the maximum AMPA set, allowing a flexibility margin of four residues to account for +/− 2 residual a.a. over the C- and *N*-termini of the AMP sequences. Since the longest AMPA predicted set was 43 a.a., the resulting maximum length cut-off was set at 47 a.a. residues. The filtered set was screened against the AMPA peptide set in order to identify matching sequences. In the spirit of simplicity, we allowed only 100% matching in the overlapping peptide sequences.

Finally, the selected set of matching peptides was screened to identify sequence patterns for the remaining enzymes: pepsin pH > 2 (Pb), trypsin (T), chymotrypsin (CT), enterokinase (E) and thrombin (Th). The total number of identified cleavage sites was recorded for peptide stability assessment.

### 2.4. Stability Assessment

The selected AMP set was assessed for stability following two scoring approaches:

(A) A cleavage stability score (CSS) was calculated for each sequence as a function of the total number of cleavage sites hydrolysed by the remaining GI tract proteases. Cleavage site recognition was performed as described above. The CSS score for peptide *x* was calculated using Equation (2):

(2) $$CSS_{x}=\frac{100}{1+\sum_{i}N_{x}^{i}},\;\;\;i\in \left\{Pb,CT,E,T,Th\right\}$$

where *N_x_^i^* is the number of identified cleavage positions specific for protease *i* in the peptide sequence *x*. The *CSS* values ranges between 100 and 0 for a sum of *N^i^* from 0 to infinity.

(B) The selected AMP set was ran against the peptide HLP [19] (http://crdd.osdd.net/raghava/hlp/index.html) using HLP’s default SVM model in order to obtain an estimation of each peptide’s half-life (*τ_x_*). The peptide set was run as subsets of equal-length peptides using the corresponding peptide length value of the tool. The HLP models were reported to have been trained on datasets pertaining to intestine-like conditions. For each peptide, the corresponding decay rate was calculated according to Equation (3):

(3) $$d_{x}=\frac{\ln\left(2\right)}{\tau_{x}}$$

### 2.5. AMP Ranking

The final AMP set was ranked on the basis of the CAS defined as:

(4) $$CAS_{x}=\frac{C\bar{S}S_{x}}{P\bar{V}x\cdot {\bar{d}}_{x}}$$

where $C\bar{S}S_{x}$, $P\bar{V}x$ and ${\bar{d}}_{x}$ represent the normalised variables by their respective maximum observed value for cleavage stability score, the AMPA propensity value and the HLP decay rate, respectively.

Finally, we analysed a set of the top 100 AMPs with respect to CAS, which ranged from 0.22 up to 1.03 for the top scorer. The choice of the top 100 AMPs does not reflect any particular scoring criterion or physical meaning and only serves the purpose of demonstrating the properties of the highest-ranking subset of AMPs in this work. When using the proposed workflow for future experimental or theoretical work, one can adjust this cutoff to obtain a broader or narrower subset.

### 2.6. Informatics

All parsing and analyses were performed in R v3.5.2. [23] using in-house developed scripts, with the exception of the AMPA and HLP runs. Additionally, a number of publicly available R packages were used in the various scripts. These include stringr [26], eulerr [27], ggpubr [28], ggplot2 [29], igraph [30] and dplyr [31].

### 2.7. Physicochemical Properties

All physicochemical properties of the AMP set were determined by running the peptides on the HLP [19] and CellPPD [21] tools. Supplemental Table S2 contains the relevant values for each peptide, while summary statistics and distributions are given in Supplemental Table S1 and Figure S2, respectively.

## 3. Results

Running all five proteomes shown in Figure 1A (totalling 1665 unique protein sequences) in AMPA returned, as shown in Figure 1B, a total of 3285 stretches with predicted antimicrobial properties, from which 2506 were unique across all proteomes. As expected, the milk whey proteomes from Chios and *C. prisca* returned the highest number (~1300) of predicted AMPs, since their proteomes have the highest number of identified proteins. On the contrary, the feta proteome returned the smallest set, comprising 861 AMPs.

The same protein sequences were digested in silico as described in Methods (Section 2). Proteins are exposed to different proteases during digestion along the GI tract, with pepsin in acidic stomach conditions (pH < 1.8, Pa) acting before the proteases present in the duodenum and intestinal tract, such as Pb (pH > 2.0), CT, T and E, as well as the proteases of microbial origin or in located in the blood, such as T. In order to adhere to and approximate the above spatiotemporal separation between protease activities, we initially extracted all possible peptides assuming complete (i.e., Pa hydrolyses all Pa-specific cleavage positions) and partial (i.e., only some Pa-specific cleavage positions are hydrolysed) pepsin digestion. For cleavage following pepsin exposure, we considered only endogenous proteases (CT, T, Pb, E and Th) and, in the spirit of simplicity, we omitted microbial proteases like Arg-C proteinase and Asp-N endopeptidase.

**Figure 2:**
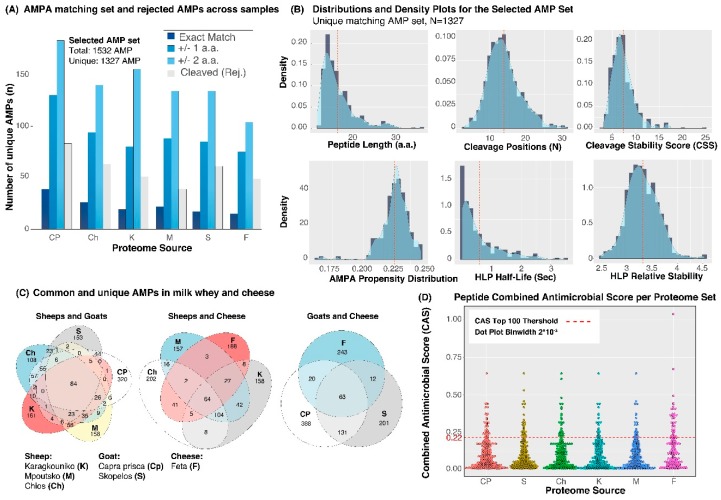
(**A**) Barplot showing the number of selected matching AMPs from the AMPA and pepsin digestion sets and the rejected peptides per proteome, grouped on the basis of the length of residual amino acids upstream and downstream of the corresponding AMPA peptide’s C- and N-termini. (**B**) Histogram and density plot of the distribution of various features of the selected AMP set. These include the peptide sequence length, number of cleavage positions by intestinal proteases, resultant stability score, AMPA propensity score, HLP half-life and HLP relative stability. The red dashed line corresponds to the mean value. (**C**) Venn diagrams showing the number of common and unique AMPs across different proteome sets. (**D**) Dotplot of the combined antimicrobial score across proteomes and the top 100 in rank over a combined antimicrobial score (CAS) threshold of 0.22.

As shown in Figure 2A and Table 1, the resultant pepsin-digested set screened against the AMPA-identified set returned 1327 unique matching sequences out of a total of 1532 sequences. The latter set did not include the matching digested peptides with lengths outside the selection range [12–47 a.a. residues] or peptides with residual sequences upstream and downstream of the N- and C-termini over 2 a.a.-long. While this threshold was set arbitrarily, we empirically found a reasonable balance between under- and over-represented AMP peptides in the datasets. Furthermore, the set tested in CellPPD [21,22] confirmed that approximately 95% of the set was predicted to be able to penetrate membranes (Supplemental Information Figure S1). The physicochemical characteristics of the predicted antimicrobial peptides given in Supplemental Information (Table S1 per peptide, summary statistics in Table S1 and distributions in Figure S2) showed an average length of 18 a.a., an amphipathicity index of 0.77, a net charge of +3.6, a hydrophobicity index of −0.17, an isoelectric point (pI) of 10.34 and a molecular weight of 2.1 kDa.

Overall, approximately 80% of the AMPA-predicted AMPs were rejected since pepsin cleavage sites were found at positions within or over 2 a.a. upstream and/or downstream of the target sequence. The selected set of the 1327 AMPs derived from pepsin digestion comprised 83 exact matches, i.e., the pepsin cleavage positions matched the starting and ending a.a. residue from the AMPA prediction, while the remaining set was cleaved at one to two residues over the starting or ending positions.

The selected set of 1327 AMPs was back-traced across the original proteome sets as shown in Figure 2A. Interestingly, the order of the number of selected AMPs did not follow the size of the proteome for all breeds, as shown in Table 1. For example, the CP milk proteome produced approximately 602 AMPs from 595 protein sequences, while the Ch proteome, which was the largest set (685 sequences, ratio = 1.01), ranked lower with approximately 407 selected AMPs and a ratio of 0.65. On the other hand, the feta cheese proteome produced the lowest number of matching AMPs, while indeed being the smallest proteome. The various features of the population of the selected AMP set followed skewed normal distributions, as shown in Figure 2B, for number of cleavage sites (non-pepsin-specific), CSS, AMPA propensity score and HLP relative stability score, while peptide length and half-life reflected a log normal distribution.

Comparing the sheep- and goat-milk-derived proteomes shown in Figure 2C, we identified 84 AMPs that are common across all animal breeds, while CP milk proteome presented the highest number of unique AMPs (~320). The feta cheese proteome was predicted to have 64 and 63 AMPs in common with the three sheep and goat breeds’ proteomes, respectively, while unique AMPs were overrepresented in feta considering its small proteome size relative to the other sets.

**Figure 3:**
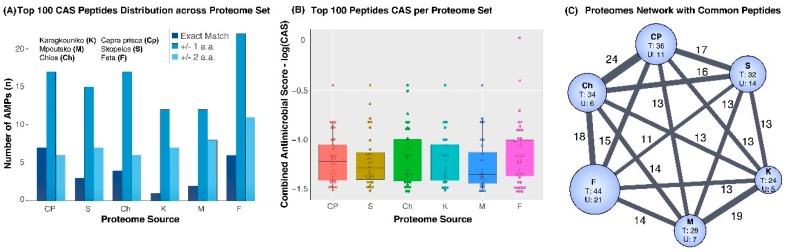
Analysis of the top 100 ranking AMPs. (**A**) Barplot showing the number of AMP from the top 100 set, across proteomes. (**B**) Dot and box plots showing the CAS score across proteomes. (**C**) Network of total, common and unique AMPs across proteomes. Nodes represent proteome sets with a size proportional to the total number of AMPs identified in each proteome. U represents the number of unique AMPs in each proteome, while edge size is proportional to the number of common AMPs shown as edge label. CP: *Capra prisca*, S: Skopelos, K: Karagkouniko, M: Mpoutsko, Ch: Chios, F: feta cheese.

Ranking the selected AMP set on the basis of the CAS and selecting the top 100 AMP peptides revealed an interesting imbalance in their representation across proteomes. Their population metrics are given in Table 2. Figure 3A shows that 36 top AMPs were traced in CP, 34 in Ch and <33 in the remaining animal species. Worth noting, the highest number of top AMP (44) were traced in feta cheese, from which 21 were not found in any of the other proteomes, albeit feta cheese having the smallest proteome size. The CAS score boxplots in Figure 3B show that F followed by Ch have the highest share of the top 100 AMP set and antimicrobial potential relative to the other proteomes analysed in this work. The top 100 AMP set is given in Supplemental Table S2 (top 100 entries) and summarised in the network shown in Figure 3C.

## 4. Discussion

The milk whey from sheep and goat breeds [15] and a specific fermentation dairy product, i.e., feta cheese [18] were found from our analysis to comprise a rich source of proteins with antimicrobial traits [2,3]. More importantly, several peptides derived from protein digestion, early along the GI tract, matched the sequences predicted by AMPA with the aforementioned antimicrobial traits. This suggests that the peptides resulting from milk digestion can potentially have a modulatory effect on the human gut microbiome profile [5,6]. Comparing the physicochemical properties (given in Supplemental Information) with those in several publicly available AMP databases, the selected AMP set in this work showed agreement with similar distributions reported in several databases containing experimentally validated AMPs, such as dbAMP [32], DBAASP [33], APD [34], CAMP [35] and LAMP [36]. The physicochemical properties were further evaluated by analysing the AMPs found in DBAASP, which contains the highest number of entries. Supplemental Table S1 shows that the DBAASP values approximate the corresponding values of the selected AMP set in this work. Finally, nearly 95% of the AMP set was predicted to have cell-penetrating ability by CellPPD [21].

In this work, we considered that the magnitude of the antimicrobial effect of a given peptide can be approximated as a function of two factors: (a) The antimicrobial propensity emerging by its amino acid physicochemical characteristics, i.e., the ability to either penetrate membrane bilayers and/or modulate host immune responses and (b) Its bioavailability which is proportional to its resistance to proteolysis within the compartment of interest. The former was derived from the AMPA antimicrobial peptide predictor [10,11], while the latter was quantified with respect to the peptides’ affinity to endogenous proteases. Yet, the amount of cleavage recognition patterns in a given peptide sequence is only one factor in a more complex scheme that determines its actual decay rate reflecting the differential stability of peptides with different amino acid composition and different biological behaviours [37,38,39]. In order to approximate a more accurate estimation, we also incorporated HLP [19] in our ranking, a peptide half-life prediction model trained on peptide decay data from crude intestine extracts. These metrics allowed us to reach a relative assessment of the proteomes under study for the AMP set of interest rather than a physical quantification of AMP properties which was out of the scope of this work.

Our results suggest that the diversity of the proteome does not necessarily correlate with the AMP diversity that can actually occur via protease digestion. Also, some AMPs which scored low in antimicrobial propensity did not necessarily ranked high with respect to CAS, since they were predicted to be more susceptible to rapid proteolysis. More specifically, the AMP predicted with the highest antimicrobial potential, i.e., the lowest propensity (FHKFICKMMKIYL) ranked only at the 965th CAS position due to a high predicted decay rate (*d_529_21_* = 1.868 s^−1^) and a CSS score (6.67) slightly lower than the mean.

Comparing the milk whey from the animal breeds of interest, we observed that the two goat breeds (Skopelos and *C. prisca*) showed higher AMP-to-proteome size ratios than the sheep breeds, but these differences were not statistically significant in Kruskal–Wallis non-parametric tests. Feta cheese returned a relatively low number of selected AMPs but surprisingly it resulted to be the most represented proteome in the top 100 AMP set which comprises the AMPs with the highest antimicrobial effect and resistance to proteolysis. Since feta cheese is produced using milk from the goat and sheep breeds discussed above, an interesting future research avenue will be to decipher whether this bias in more stable AMPs is introduced during the fermentation process and which mechanisms are responsible for it. Recent work has suggested that lactic acid microbes have a central role in the release of encrypted bioactive peptides during this process [40].

Finally, this work aimed at profiling the diverse range of AMPs that can occur and be active within the GI tract. We followed the rational that exposure of whole proteins to gastric pepsin precedes proteolysis from other proteases, therefore, peptides produced by pepsin digestion are predominant and more likely to occur. Yet, under conditions of incomplete pepsin digestion, a broader diversity of active AMPs can be produced as a result of digestion from the other endogenous or bacterial proteases. Future research can focus on the top predicted AMPs to determine experimentally their antimicrobial activity and degradation rate under intestine or intestine-like conditions. Simultaneously, an intriguing prospective will be to employ more sophisticated protease cleavage models as well as quantitative proteomics data in order to predict a range of AMPs concentration with respect to the relative abundance of their parent proteins. Under ideal conditions and given sufficient time, all proteins can be fully degraded through hydrolysis by endogenous proteases and proteases from commensal microbes. Nevertheless, during this dynamic process, it is expected that some peptides will be stable enough to exert temporarily their effects. Incorporating enzyme kinetics to model dynamically the cleavage activity of each type of protease can aid towards shedding light on these dynamics under intestine-relevant conditions. Such approaches have already being demonstrated with promising results [41,42].

We anticipate that adapting and employing this workflow to obtain AMP profiling in other functional foods, but also extending it to probe for other types of bioactive peptides, can shape a better understanding of the complex interaction landscape between the host, its microbiome and its dietary habits. Finally, the workflow we employed, allowing fast screening of entire proteomes for antimicrobial peptides that can occur during digestion, can assist the ongoing effort to design peptides as medicinal products which can be efficiently delivered through the oral route [39].

## Figures and Tables

**Table 1:** Population metrics for the selected AMP set.

Proteome	Proteins (n)	AMPs (n)	Ratio to Proteome	Mean Propensity	Mean *d_x_* (s^−1^)	Mean CSS	Mean CAS
**CP**	595	602	1.012	0.224	1.270	7.08	0.062
**S**	486	407	0.837	0.224	1.155	7.156	0.069
**Ch**	685	442	0.645	0.225	1.120	6.891	0.069
**K**	550	416	0.756	0.225	1.238	6.989	0.063
**M**	583	415	0.712	0.224	1.199	6.869	0.064
**F**	489	338	0.691	0.224	0.968	7.382	0.086

**Table 2:** Population metrics for the top 100 AMP (CAS > 0.22).

Proteome	Proteins (n)	AMPs (n)	Ratio to proteome	Mean Propensity	Mean *d_x_* (s^−1^)	Mean CSS	Mean CAS
**CP**	595	36	0.061	0.229	0.288	9.895	0.312
**S**	486	32	0.066	0.231	0.310	10.31	0.311
**Ch**	685	34	0.050	0.229	0.283	9.713	0.326
**K**	550	24	0.044	0.231	0.301	10.304	0.311
**M**	583	28	0.048	0.228	0.296	9.632	0.3
**F**	489	44	0.090	0.229	0.271	10.045	0.338

## Supplementary Materials

The following are available online: https://www.mdpi.com/2227-7382/7/4/32/s1. Supplemental Information: Document containing Supplemental Figure S1 with the prediction summary of CellPPD, Supplemental Figure S2 with the distributions of selected physicochemical characteristics of the dataset and Supplemental Table S1 with the summary of the physicochemical characteristics of the AMP set. Supplemental Table S2: CSV file containing the set of selected AMPs resulting from pepsin digestion and matching the AMPA-predicted sequences. The spreadsheet provides for each AMP all the information regarding the originating protein sequence, proteomes in which it was identified, AMPA and HLP variables along with the estimated cleavage stability and combined antimicrobial score.

## Availability of Resources

All R scripts developed and used in this work are available upon request.

## References

[1] Pellegrini A. (2005). Antimicrobial Peptides from Food Proteins. Curr. Pharm. Des..

[2] Ng T.B., Wong J.H., Almahdy O., El-Fakharany E.M., El-Dabaa E., Redwan E.R.M. (2012). Antimicrobial activities of casein and other milk proteins. Casein: Production, Uses and Health Effects.

[3] Haque E., Chand R., Kapila S. (2009). Biofunctional properties of bioactive peptides of milk origin. Food Rev. Int..

[4] Miner-Williams W.M., Stevens B.R., Moughan P.J. (2014). Are intact peptides absorbed from the healthy gut in the adult human?. Nutr. Res. Rev..

[5] Chen L., Li Y., Li J., Xu X., Lai R., Zou Q. (2007). An antimicrobial peptide with antimicrobial activity against Helicobacter pylori. Peptides.

[6] Dallas D.C., Guerrero A., Khaldi N., Castillo P.A., Martin W.F., Smilowitz J.T., Bevins C.L., Barile D., German J.B., Lebrilla C.B. (2013). Extensive in vivo human milk peptidomics reveals specific proteolysis yielding protective antimicrobial peptides. J. Proteome Res..

[7] Wang S., Zeng X., Yang Q., Qiao S. (2016). Antimicrobial peptides as potential alternatives to antibiotics in food animal industry. Int. J. Mol. Sci..

[8] Kumar P., Kizhakkedathu J., Straus S. (2018). Antimicrobial Peptides: Diversity, Mechanism of Action and Strategies to Improve the Activity and Biocompatibility In Vivo. Biomolecules.

[9] Ulm H., Wilmes M., Shai Y., Sahl H.G. (2012). Antimicrobial host defensins specific antibiotic activities and innate defense modulation. Front. Immunol..

[10] Torrent M., Di Tommaso P., Pulido D., Nogués M.V., Notredame C., Boix E., Andreu D. (2012). AMPA: An automated web server for prediction of protein antimicrobial regions. Bioinformatics.

[11] Torrent M., Nogués V.M., Boix E. (2009). A theoretical approach to spot active regions in antimicrobial proteins. BMC Bioinforma..

[12] Fadaei V. (2012). Milk Proteins-derived antibacterial peptides as novel functional food ingredients. Ann. Biol. Res..

[13] Atanasova J., Ivanova I. (2010). Antibacterial peptides from goat and sheep milk proteins. Biotechnol. Biotechnol. Equip..

[14] Park Y.W., Nam M.S. (2015). Bioactive Peptides in Milk and Dairy Products: A Review. Korean J. Food Sci. Anim. Resour..

[15] Anagnostopoulos A.K., Katsafadou A.I., Pierros V., Kontopodis E., Fthenakis G.C., Arsenos G., Karkabounas S.C., Tzora A., Skoufos I., Tsangaris G.T. (2016). Milk of Greek sheep and goat breeds; characterization by means of proteomics. J. Proteomics.

[16] Brandelli A., Daroit D.J., Corrêa A.P.F. (2015). Whey as a source of peptides with remarkable biological activities. Food Res. Int..

[17] Hati S., Patel N., Sakure A., Mandal S. (2018). Influence of Whey Protein Concentrate on the Production of Antibacterial Peptides Derived from Fermented Milk by Lactic Acid Bacteria. Int. J. Pept. Res. Ther..

[18] Anagnostopoulos A.K., Tsangaris G.T. (2018). Feta cheese proteins: Manifesting the identity of Greece’s National Treasure. Data Br..

[19] Sharma A., Singla D., Rashid M., Raghava G.P.S. (2014). Designing of peptides with desired half-life in intestine-like environment. BMC Bioinforma..

[20] Bateman A. (2019). UniProt: A worldwide hub of protein knowledge. Nucleic Acids Res..

[21] Gautam A., Chaudhary K., Kumar R., Raghava G.P.S. (2015). Computer-aided virtual screening and designing of cell-penetrating peptides. Cell-Penetrating Peptides: Methods and Protocols.

[22] Gautam A., Chaudhary K., Kumar R., Sharma A., Kapoor P., Tyagi A., Raghava G.P.S. (2013). In silico approaches for designing highly effective cell penetrating peptides. J. Transl. Med..

[23] (2018). R Development Core Team R: A Language and Environment for Statistical Computing. https://cran.r-project.org.

[24] Gasteiger E., Hoogland C., Gattiker A., Duvaud S., Wilkins M.R., Appel R.D., Bairoch A. (2005). Protein Identification and Analysis Tools on the ExPASy Server. The Proteomics Protocols Handbook.

[25] Anekthanakul K., Hongsthong A., Senachak J., Ruengjitchatchawalya M. (2018). SpirPep: An in silico digestion-based platform to assist bioactive peptides discovery from a genome-wide database. BMC Bioinforma..

[26] Wickham H. (2017). R: Package ‘stringr.’ CRAN. https://cran.r-project.org/web/packages/stringr/stringr.pdf.

[27] Larsson J., Gustafsson P. A case study in fitting area-proportional euler diagrams with ellipses using eulerr. Proceedings of the CEUR Workshop Proceedings.

[28] Kassambara A. (2018). ggpubr: “ggplot2” Based Publication Ready Plots. R package version 0.1.7. https://cran.r-project.org/web/packages/ggpubr/ggpubr.pdf..

[29] Wickham H. (2011). ggplot2. Wiley Interdiscip. Rev. Comput. Stat..

[30] Hunter J.E., Cohen S.H. (2007). Package: igraph. Educ. Psychol. Meas..

[31] Wickham H., Francois R. (2016). The dplyr package. R Core Team.

[32] Jhong J.H., Chi Y.H., Li W.C., Lin T.H., Huang K.Y., Lee T.Y. (2019). DbAMP: An integrated resource for exploring antimicrobial peptides with functional activities and physicochemical properties on transcriptome and proteome data. Nucleic Acids Res..

[33] Pirtskhalava M., Gabrielian A., Cruz P., Griggs H.L., Squires R.B., Hurt D.E., Grigolava M., Chubinidze M., Gogoladze G., Vishnepolsky B. (2016). DBAASP v.2: An enhanced database of structure and antimicrobial/cytotoxic activity of natural and synthetic peptides. Nucleic Acids Res..

[34] Wang Z. (2004). APD: the Antimicrobial Peptide Database. Nucleic Acids Res..

[35] Waghu F.H., Gopi L., Barai R.S., Ramteke P., Nizami B., Idicula-Thomas S. (2014). CAMP: Collection of sequences and structures of antimicrobial peptides. Nucleic Acids Res..

[36] Zhao X., Wu H., Lu H., Li G., Huang Q. (2013). LAMP: A Database Linking Antimicrobial Peptides. PLoS ONE.

[37] Boöttger R., Hoffmann R., Knappe D. (2017). Differential stability of therapeutic peptides with different proteolytic cleavage sites in blood, plasma and serum. PLoS ONE.

[38] Naimi S., Zirah S., Hammami R., Fernandez B., Rebuffat S., Fliss I. (2018). Fate and biological activity of the antimicrobial lasso peptide microcin J25 under gastrointestinal tract conditions. Front. Microbiol..

[39] Renukuntla J., Vadlapudi A.D., Patel A., Boddu S.H.S., Mitra A.K. (2013). Approaches for enhancing oral bioavailability of peptides and proteins. Int. J. Pharm..

[40] Pessione E., Cirrincione S. (2016). Bioactive molecules released in food by lactic acid bacteria: Encrypted peptides and biogenic amines. Front. Microbiol..

[41] Deng Z., Mao J., Wang Y., Zou H., Ye M. (2017). Enzyme Kinetics for Complex System Enables Accurate Determination of Specificity Constants of Numerous Substrates in a Mixture by Proteomics Platform. Mol. Cell. Proteomics.

[42] Gorris H.H., Bade S., Röckendorf N., Albers E., Schmidt M.A., Fránek M., Frey A. (2009). Rapid profiling of peptide stability in proteolytic environments. Anal. Chem..

