# Supplementary material for: In Silico Identification of Antimicrobial Peptides in the Proteomes of Goat and Sheep Milk and Feta Cheese
Source: Proteomes. 2019 Sep 21;7(4):32. doi: 10.3390/proteomes7040032 (PMC6958355; doi:10.3390/proteomes7040032)
Supplement: Supplementary file 1 [file proteomes-07-00032-s001.zip › Suppl-580463/Supplemental Information.pdf]

In silico identification of antimicrobial peptides in goat, sheep milk and feta cheese proteomes

Supplemental Information

S1. CellPPD Prediction Results

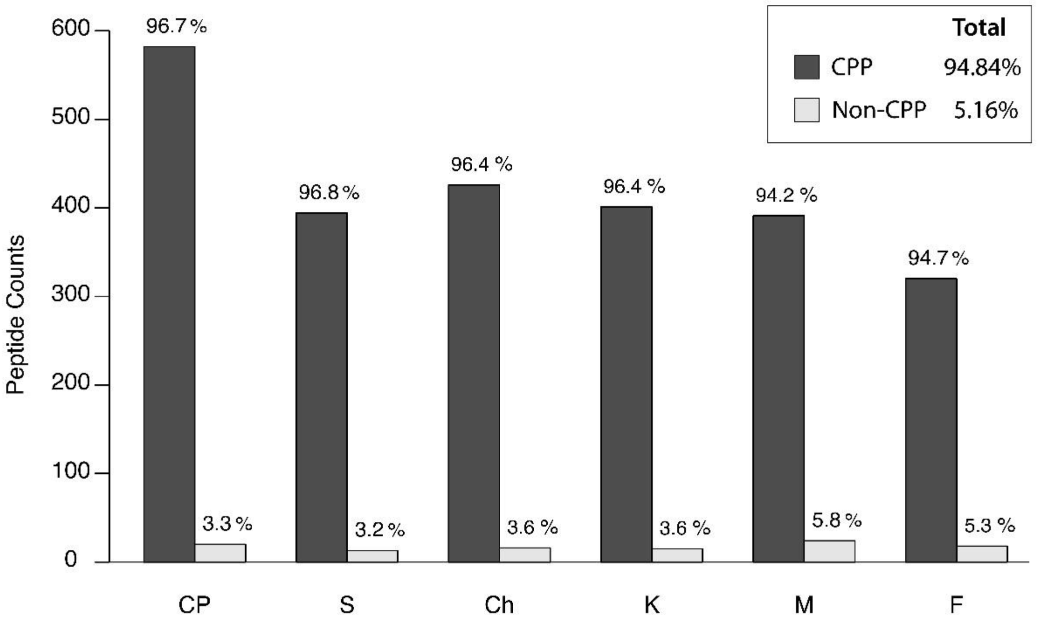

Figure S1. Barplot showing the number of AMPs selected after pepsin digestion that are predicted by CellPPD[1,2] to be cell penetrating (CPP) or not (non-CPP). The bars are grouped by proteome source and percentages show the CPP to non-CPP percentage per proteome.

S2. Physicochemical Properties of the selected AMP set

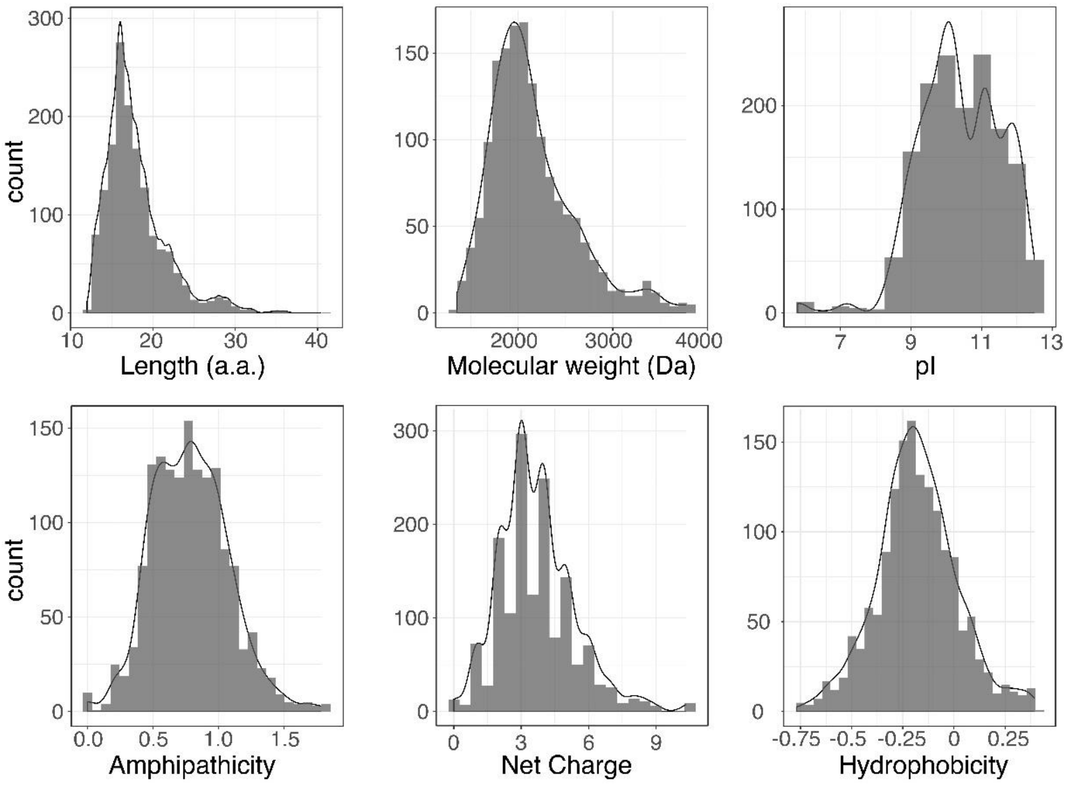

Figure S2. Histograms and density plots for selected physicochemical characteristics. Length is given as number of a.a. residues. Molecular weight, isoelectric point (pI), Amphipathicity, Charge and Hydrophobicity values were obtained by running the AMP set in CellPPD.

Table S1. The Summary Statistics (Mean value, Standard Deviation and Range) of physicochemical values per proteome source and comparison with DBAASP.

	CP	S	Ch	K	M	F	All	DBAASP*
				**Length (a.a.)**				
Mean (SD)	17.70 (± 3.589)	17.81 (± 3.637)	17.53 (± 3.588)	17.93 (± 3.643)	18.03 (± 3.908)	17.77 (± 3.932)	**17.79(± 3.702)**	**19.71(± 13.764)**
Range	13, 36	13, 41	13, 36	12, 36	13, 36	12, 36	**12, 41**	**1, 190**
				**Amphipathicity**				
Mean (SD)	0.75 (± 0.270)	0.78 (± 0.293)	0.76 (± 0.270)	0.79 (± 0.311)	0.76 (± 0.294)	0.80 (± 0.286)	**0.77 (± 0.287)**	**0.59 (± 0.196)**
Range	0.00, 1.62	0.00, 1.51	0.00, 1.61	0.00, 1.85	0.00, 1.77	0.16, 1.85	**0.00, 1.85**	**0.00, 1.23**
				**Net Charge**				
Mean (SD)	3.58 (± 1.654)	3.53 (± 1.731)	3.54 (± 1.556)	3.62 (± 1.575)	3.60 (± 1.842)	3.74 (± 1.820)	**3.60 (± 1.691)**	**4.25 (± 3.080)**
Range	0, 9	0, 10	0, 9	0, 9	0, 9	0.0, 10.5	**0.0, 10.5**	**0.0, 10.5**
				**Isoelectric point - pI**				
Mean (SD)	10.36 (± 1.168)	10.16 (± 1.209)	10.41 (± 1.137)	10.38 (± 1.136)	10.26 (± 1.250)	10.47 (± 1.033)	**10.34 (± 1.164)**	**10.75 (± 1.961)**
Range	5.84, 12.61	5.87, 12.71	5.87, 12.32	5.87, 12.48	5.79, 12.71	6.50, 12.31	**5.79, 12.71**	**3.16, 13.779**
				**Hydrophobicity**				
Mean (SD)	-0.164 (± 0.197)	-0.19 (± 0.204)	-0.16 (± 0.198)	-0.19 (± 0.210)	-0.16 (± 0.205)	-0.19 (± 0.189)	**-0.17 (± 0.201)**	**-0.16 (± 0.967)**
Range	-0.74, 0.40	-0.74, 0.40	-0.74, 0.40	-0.77, 0.40	-0.74, 0.40	-0.77, 0.35	**-0.77, 0.40**	**-4.5, 2.8**
				**Molecular weight (Da)**				
Mean (SD)	2133.28 (± 438.829)	2127.59 (± 403.729)	2097.67 (± 432.966)	2159.98 (± 430.373)	2158.70 (± 471.704)	2131.71 (± 508.410)	**2134.45 (± 446.363)**	**2424 (± 823.078)**
Range	1352.9, 3605.5	1398.08, 3489.53	1352.9, 3507.6	1352.90, 3545.72	1352.90, 3694.01	1352.90, 3834.07	**1352.90, 3834.07**	**1060, 5286**

*DBAASP Length was calculated using all the entries (n=13554) downloaded from the DBAASP database[3]. All other physicochemical properties were calculated on the subset of peptide monomers with length ranging between 12-43 a.a. (n=7737) in order to provide an accurate comparison with the selected AMP set identified in this work.

Supplemental References:

1. Gautam, A.; Chaudhary, K.; Kumar, R.; Sharma, A.; Kapoor, P.; Tyagi, A.; Raghava, G.P.S. In silico approaches for designing highly effective cell penetrating peptides. *J. Transl. Med.* **2013**.
2. Gautam, A.; Chaudhary, K.; Kumar, R.; Raghava, G.P.S. Computer-aided virtual screening and designing of cell-penetrating peptides. In *Cell-Penetrating Peptides: Methods and Protocols*; 2015 ISBN 9781493928064.
3. Pirtskhalava, M.; Gabrielian, A.; Cruz, P.; Griggs, H.L.; Squires, R.B.; Hurt, D.E.; Grigolava, M.; Chubinidze, M.; Gogoladze, G.; Vishnepolsky, B.; et al. DBAASP v.2: An enhanced database of structure and antimicrobial/cytotoxic activity of natural and synthetic peptides. *Nucleic Acids Res.* **2016**.
